# The CaaX specificities of Arabidopsis protein prenyltransferases explain *era1 *and *ggb *phenotypes

**DOI:** 10.1186/1471-2229-10-118

**Published:** 2010-06-18

**Authors:** Michelle Andrews, David H Huizinga, Dring N Crowell

**Affiliations:** 1 Department of Biological Sciences, Idaho State University, Pocatello, ID 83209, USA; 2 Department of Biology, Indiana University-Purdue University, Indianapolis, IN 46202, USA; 3 Dow AgroSciences LLC, Indianapolis, IN 46268, USA

## Abstract

**Background:**

Protein prenylation is a common post-translational modification in metazoans, protozoans, fungi, and plants. This modification, which mediates protein-membrane and protein-protein interactions, is characterized by the covalent attachment of a fifteen-carbon farnesyl or twenty-carbon geranylgeranyl group to the cysteine residue of a carboxyl terminal CaaX motif. In Arabidopsis, *era1 *mutants lacking protein farnesyltransferase exhibit enlarged meristems, supernumerary floral organs, an enhanced response to abscisic acid (ABA), and drought tolerance. In contrast, *ggb *mutants lacking protein geranylgeranyltransferase type 1 exhibit subtle changes in ABA and auxin responsiveness, but develop normally.

**Results:**

We have expressed recombinant Arabidopsis protein farnesyltransferase (PFT) and protein geranylgeranyltransferase type 1 (PGGT1) in *E. coli *and characterized purified enzymes with respect to kinetic constants and substrate specificities. Our results indicate that, whereas PFT exhibits little specificity for the terminal amino acid of the CaaX motif, PGGT1 exclusively prenylates CaaX proteins with a leucine in the terminal position. Moreover, we found that different substrates exhibit similar K_m _but different k_cat _values in the presence of PFT and PGGT1, indicating that substrate specificities are determined primarily by reactivity rather than binding affinity.

**Conclusions:**

The data presented here potentially explain the relatively strong phenotype of *era1 *mutants and weak phenotype of *ggb *mutants. Specifically, the substrate specificities of PFT and PGGT1 suggest that PFT can compensate for loss of PGGT1 in *ggb *mutants more effectively than PGGT1 can compensate for loss of PFT in *era1 *mutants. Moreover, our results indicate that PFT and PGGT1 substrate specificities are primarily due to differences in catalysis, rather than differences in substrate binding.

## Background

**Figure 1:**
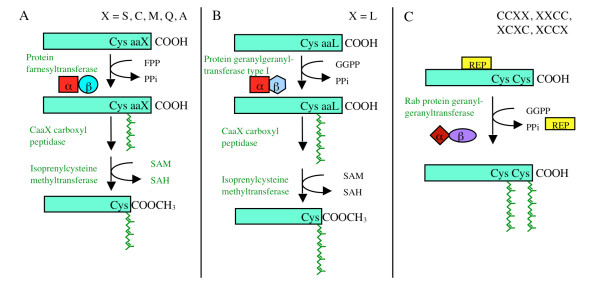
**Protein prenylation and processing in eukaryotes**. A, prenylation and processing of farnesylated CaaX proteins. B, prenylation and processing of geranylgeranylated CaaX proteins. C, prenylation of RAB GTPases.

Protein farnesylation is the process by which proteins bearing a carboxyl terminal CaaX motif (C = Cys; a = aliphatic; X = Ser, Cys, Met, Gln, Ala) are post-translationally modified by the covalent attachment of a fifteen-carbon farnesyl group [1-4]. This modification results in the formation of a stable thioether bond between the cysteine of the CaaX motif and the farnesyl moiety, with farnesyl diphosphate serving as the farnesyl donor (Figure 1). This lipidation reaction is catalyzed by protein farnesyltransferase (PFT), which is a cytosolic enzyme consisting of α- and β-subunits [1-4]. In a similar process, proteins bearing a carboxyl terminal CaaX motif with Leu, Ile, Met, or Phe in the terminal position are modified by the covalent attachment of a twenty-carbon geranylgeranyl group to the cysteine of the CaaX motif. This modification is catalyzed by protein geranylgeranyltransferase type I (PGGT1), which is a cytosolic enzyme consisting of an α-subunit identical to that of PFT and a distinct β-subunit [1-5]. A third enzyme, protein geranylgeranyltransferase type II (PGGT II), also called RAB geranylgeranyltransferase (RAB GGT), catalyzes the geranylgeranylation of RAB proteins bound to the RAB ESCORT PROTEIN (REP). All three enzymes have been found in protozoans, metazoans, fungi, and plants, including peas [6,7], tomato [8,9], and *Arabidopsis *[10-14].

In *Arabidopsis*, a single gene encodes the common α-subunit of PFT and PGGT1 (*PLURIPETALA, PLP*, At3g59380) [14], a second gene encodes the β-subunit of PFT (*ENHANCED RESPONSE TO ABA1*, *ERA1*, At5g40280) [11,13], and a third gene encodes the β-subunit of PGGT1 (*GERANYLGERANYLTRANSFERASE BETA*, *GGB*, At2g39550) [10,12]. The *ERA1 *gene was so named because knockout mutations in this gene cause an enhanced response to abscisic acid (ABA) in both seed germination and stomatal closure assays. Consequently, *era1 *mutants exhibit increased seed dormancy and stomatal closure in response to ABA, and are drought tolerant [11,13,15-17]. These observations suggest that at least one farnesylated protein functions as a negative regulator of ABA signaling. However, to date, a farnesylated negative regulator of ABA signaling has not been definitively identified*. era1 *plants also exhibit enlarged meristems and supernumerary floral organs, especially petals, and this phenotype is greatly exaggerated in *plp *mutants lacking the common α-subunit of PFT and PGGT1 [14,18-21]. The more severe developmental phenotype of *plp *mutants compared to *era1 *mutants suggests that PGGT1 partially compensates for loss of PFT in *era1 *mutants [14]. Plants with defects in the *GGB *gene exhibit increased ABA-induced stomatal closure and auxin-induced lateral root formation [12], but without significant developmental phenotypes. These observations suggest that at least one geranylgeranylated protein functions as a negative regulator of ABA signaling and at least one functions as a negative regulator of auxin signaling. Indeed, ROP2 and ROP6, which are geranylgeranylated small GTPases [22,23], have been shown to function as negative regulators of ABA signaling, and ROP2 and AUX 2-11 (a geranylgeranylated member of the AUX/IAA family) have been shown to function as negative regulators of auxin signaling [10,24]. Moreover, *Arabidopsis *plants possess two genes encoding G protein γ-subunits, both of which are geranylgeranylated, and mutants lacking either of these genes exhibit an enhanced response to auxin-induced lateral root formation [25]. Prenylated proteins have also been implicated in a plethora of other processes, including calcium signal transduction [26,27], response to heat and heavy metal stress [28-30], cytokinin biosynthesis [31], and regulation of the cell division cycle [6,32,33]. Given these multiple roles, it is surprising that, unlike other organisms, *Arabidopsis *plants survive without the shared α-subunit of PFT and PGGT1 [14].

Proteins that are prenylated by either PFT or PGGT1 are further modified. First, the aaX portion of the CaaX motif is proteolytically removed by specific CaaX proteases (*AtSTE24*, At4g01320 and *AtFACE-2*, At2g36305 in *Arabidopsis*) [34-36] and, second, the isoprenylcysteine at the newly formed carboxyl terminus is methylated (Figure 1) [37-43]. Two distinct isoprenylcysteine methyltransferase (ICMT) enzymes, encoded by the *AtSTE14A *(At5g23320) and *AtSTE14B *(*ICMT*, At5g08335) genes, catalyze the methylation of carboxyl terminal isoprenylcysteines in *Arabidopsis *[41,43-46]. Demethylation of isoprenylcysteine methyl esters is catalyzed by isoprenylcysteine methylesterase (ICME), which is encoded by the *ICME *gene (At5g15860) [46,47].

As described above, two geranylgeranylated proteins (ROP2 and ROP6) and at least one farnesylated protein negatively regulate ABA signaling in *Arabidopsis*. However, it is not clear at the present time how these proteins function in ABA signaling. The stomata of *ggb *plants were found to exhibit an enhanced response to ABA, consistent with the known role of ROP6 in negative regulation of ABA-induced stomatal closure [12,23], but the response of *ggb *seeds to ABA was normal, despite a report that ROP2 is involved in negative regulation of ABA signaling in seeds [22]. While this may seem like a contradiction, it is possible that PFT activity in *ggb *plants is sufficient for the prenylation and function of certain prenylated proteins, such as ROP2 (i.e., PFT compensates for loss of PGGT1 in *ggb *mutants). Indeed, numerous reports exist of prenylated proteins that are substrates of both PFT and PGGT1 and others that are substrates of either PFT or PGGT1 [48,49]. Given this heterogeneity in the specificity of PFT and PGGT1 for certain CaaX proteins, deconvoluting the complex roles of protein prenylation in negative regulation of ABA signaling, meristem development, and other fundamental processes poses a significant challenge. Nevertheless, to address this problem, we characterized Arabidopsis PFT and PGGT1 with respect to substrate specificity and catalysis. These studies were aimed at answering the following questions: 1) What distinguishes plant CaaX prenyltransferases from animal and fungal prenyltransferases and what gives them their unique substrate specificities? 2) Do the substrate specificities of Arabidopsis PFT and PGGT1 potentially explain the phenotypes of *era1 *and *ggb *mutants? The results reported here indicate that Arabidopsis PFT exhibits less specificity for the terminal position of the CaaX motif than PFT enzymes from metazoans and yeast and Arabidopsis PGGT1 exhibits greater specificity for CaaX motifs with leucine in the terminal position than PGGT1 enzymes from metazoans and yeast. These results potentially explain the phenotypes of *era1 *and *ggb *mutants. Moreover, we show that different CaaX substrates exhibit differences in reactivity rather than differences in affinity in the presence of Arabidopsis PFT and PGGT1.

## Results

### Recombinant *Arabidopsis *PFT is more specific for isoprenoid substrates than PGGT1, whereas PGGT1 is more specific for CaaX substrates

**Figure 2:**
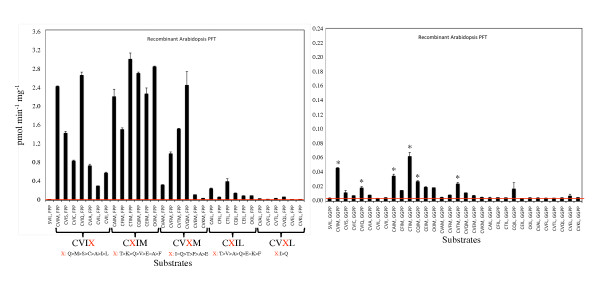
**Substrate specificity of recombinant Arabidopsis PFT**. Quantitative filter assay data are shown for recombinant Arabidopsis PFT in the presence of [1-^3^H]FPP, [1-^3^H]GGPP, and 32 distinct GFP-BD-CaaX substrates. The CaaX substrates are grouped with the variable amino acid indicated in red. Asterisks indicate PFT-catalyzed protein geranylgeranylation detectable after 4 days by SDS-PAGE and fluorography. The red line indicates background. The standard error of the mean is shown.

**Figure 3:**
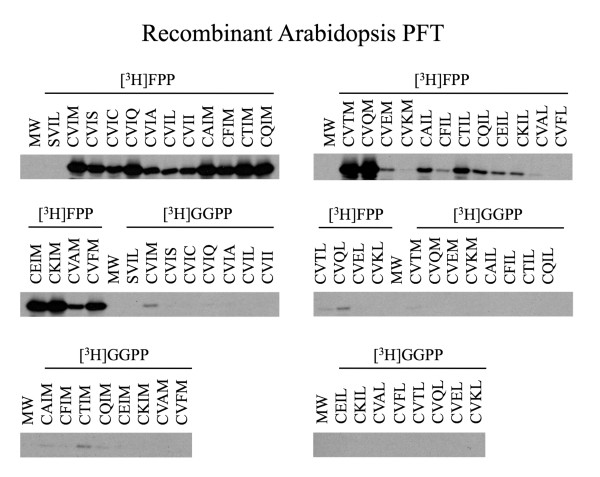
**Radiofluorograms of prenylation assays performed in the presence of recombinant Arabidopsis PFT**. Radiofluorograms corresponding to the quantitative filter assay data in Figure 2 are shown.

To functionally characterize *Arabidopsis *PFT and PGGT1, we co-expressed the *PLP *and *ERA1 *coding sequences in *E. coli *using the pETDuet-1 vector (*PLP *was expressed with an amino terminal FLAG tag and *ERA1 *was expressed with an amino terminal 6 × His tag). We also co-expressed the *PLP *and *GGB *coding sequences in *E. coli *(*PLP *was expressed with an amino terminal FLAG tag and *GGB *was expressed with an amino terminal 6 × His tag). IPTG-inducible PFT and PGGT1 activities were detected in *E. coli *extracts and analyzed for substrate specificity using [1-^3^H]FPP, [1-^3^H]GGPP, and 32 distinct GFP-BD-CaaX protein substrates, which were generated by site-directed mutagenesis of the GFP-BD-CaaX constructs recently reported by Gerber et al. (each protein substrate consists of GFP fused to the carboxyl terminal basic domain of the rice CaM61 protein and one of 32 different CaaX motifs) [50]. As shown in Figures 2 and 3, recombinant *Arabidopsis *PFT exhibited modest selectively for the terminal amino acid of the Ca_1_a_2_X motif. GFP-BD-CaaX substrates with glutamine, methionine, serine, cysteine, alanine, isoleucine, and even leucine (in descending order) were appreciably farnesylated by *Arabidopsis *PFT. As previously reported, PFT exhibited low selectivity for the a_1 _position of the Ca_1_a_2_X motif, consistent with the observation that the a_1 _position is solvent exposed and not constrained by active site amino acids [51-55]. In contrast, PFT exhibited high selectivity for the a_2 _position, with charged amino acids (basic as well as acidic) strongly excluded. GFP-BD-CaaX substrates that were efficiently farnesylated were also weakly geranylgeranylated by *Arabidopsis *PFT, but GFP-BD-CaaX farnesylation was 50-fold greater than geranylgeranylation (i.e., the y-axes in the two graphs of Figure 2 are not the same).

**Figure 4:**
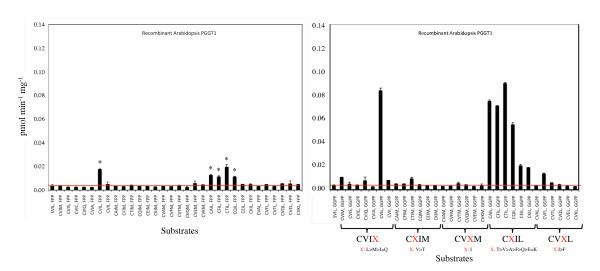
**Substrate specificity of recombinant Arabidopsis PGGT I**. Quantitative filter assay data are shown for recombinant Arabidopsis PGGT I in the presence of [1-^3^H]FPP, [1-^3^H]GGPP, and 32 distinct CaaX substrates. The CaaX substrates are grouped with the variable amino acid indicated in red. Asterisks indicate PGGT1-catalyzed protein farnesylation detectable after 4 days by SDS-PAGE and fluorography. The red line indicates background. The standard error of the mean is shown.

**Figure 5:**
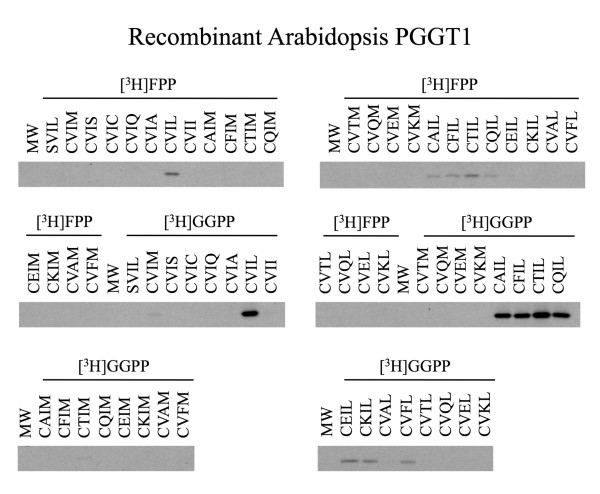
**Radiofluorograms of prenylation assays performed in the presence of recombinant Arabidopsis PGGT1**. Radiofluorograms corresponding to the quantitative filter assay data in Figure 4 are shown.

As shown in Figures 4 and 5, recombinant *Arabidopsis *PGGT1 exhibited high selectivity for the terminal amino acid of the Ca_1_a_2_X motif. Only GFP-BD-CaaX substrates ending in leucine were significantly prenylated by this enzyme (not even CVII, which is a good substrate for mammalian PGGT1, was appreciably prenylated by *Arabidopsis *PGGT1). As with PFT, PGGT1 exhibited low selectivity for the a_1 _position of the Ca_1_a_2_X motif, consistent with the observation that the a_1 _position is solvent exposed and not constrained by active site amino acids. On the other hand, PGGT1 exhibited extremely high selectivity for the a_2 _position, and only GFP-BD-CaaX substrates with a hydrophobic amino acid at the a_2 _position (CVIL and CVFL) were prenylated. GFP-BD-CaaX substrates that were efficiently geranylgeranylated were also farnesylated by PGGT1. Indeed, GFP-BD-CaaX geranylgeranylation was only 4-fold greater than farnesylation in the presence of recombinant *Arabidopsis *PGGT1.

### Purified recombinant PFT is more active than purified recombinant PGGT1

**Figure 6:**
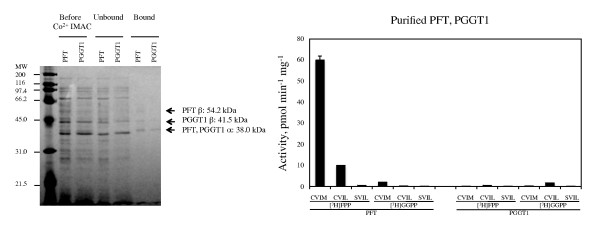
**Activity and substrate specificity of IMAC-purified recombinant Arabidopsis PFT and PGGT I**. Left Panel: *E. coli *extracts before Co^2+^-IMAC, unbound proteins, and bound (IMAC-purified) proteins are shown. An *E. coli *protein at 42 kDa, which co-migrates with the PGGT I β-subunit is present at low levels in the purified PFT and PGGT I samples. Right Panel: quantitative activity data for purified PFT and PGGT I. The standard error of the mean is shown.

The next step in the characterization of *Arabidopsis *PFT and PGGT1 was to examine the activity and substrate specificity of purified recombinant enzymes. The enzymes described above were purified by immobilized metal affinity chromatography (IMAC) using Talon^®; ^Co^2+^-based resin. As shown in Figure 6, IMAC-purified enzymes (80-90% pure) were assayed and found to exhibit the same substrate specificities as described above (PFT exhibits low specificity for the terminal amino acid of the Ca_1_a_2_X motif, whereas PGGT1 is highly selective for GFP-BD-CaaX substrates ending in leucine). Comparing purified recombinant PFT and PGGT1 allowed us to make the following conclusion: purified recombinant PFT is 30- to 100-fold more active than purified recombinant PGGT1. This is not due to errors in the expressed sequences, nor is it likely to be due to differential effects of the FLAG tag on the alpha subunit or the 6 × His-tags on the two β-subunits because the amino termini of prenyltransferase α- and β-subunits are solvent exposed and not involved in the formation or stabilization of active heterodimers [56,57]. Moreover, plant extracts (tobacco BY2 as well as *Arabidopsis *extracts) consistently exhibit 30-100 fold higher PFT activity compared with PGGT1 activity [12].

### Kinetic Analysis of Recombinant Arabidopsis PFT and PGGT1

**Figure 7:**
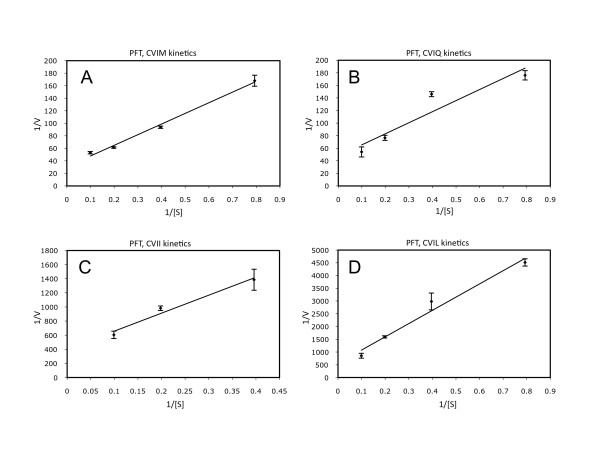
**Lineweaver-Burk plots for purified PFT**. A, GFP-BD-CVIM and [1-^3^H]FPP were used as substrates. B, GFP-BD-CVIQ and [1-^3^H]FPP were used as substrates. C, GFP-BD-CVII and [1-^3^H]FPP were used as substrates. D, GFP-BD-CVIL and [1-^3^H]FPP were used as substrates. The standard error of the mean is shown.

**Figure 8:**
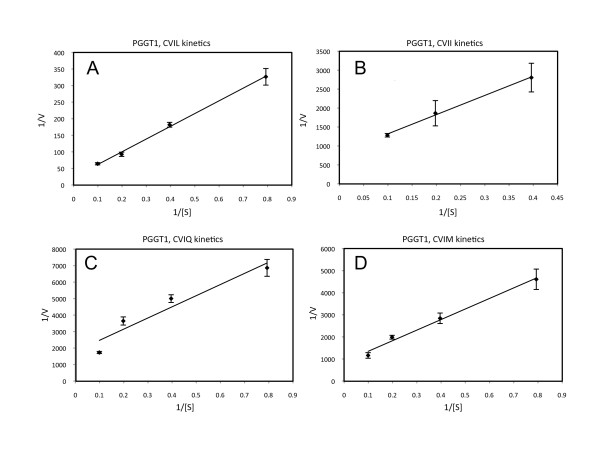
**Lineweaver-Burk plots for purified PGGT1**. A, GFP-BD-CVIL and [1-^3^H]GGPP were used as substrates. B, GFP-BD-CVII and [1-^3^H]GGPP were used as substrates. C, GFP-BD-CVIQ and [1-^3^H]GGPP were used as substrates. D, GFP-BD-CVIM and [1-^3^H]GGPP were used as substrates. The standard error of the mean is shown.

Purified recombinant Arabidopsis PFT and PGGT1 were subjected to kinetic analyses under Michaelis-Menten conditions (product formation was linear with time and substrate conversion was less than 10%). The results of these experiments were interpreted by Lineweaver-Burk analysis and are shown in Figures 7 and 8. The catalytic constants (k_cat_/K_m_) shown in Table 1 for Arabidopsis PFT confirm the results shown in Figures 2 and 3. Both data sets demonstrate the following substrate preferences for Arabidopsis PFT (normalized to 1.0 for GFP-BD-CVIQ): GFP-BD-CVIQ (1.0), GFP-BD-CVIM (0.60), GFP-BD-CVII (0.21) and GFP-BD-CVIL (0.06). Moreover, the results in Table 1 show that, while different CaaX substrates (CVIQ, CVIM, CVII, and CVIL) have similar K_m _values, they have markedly different k_cat _values in the presence of Arabidopsis PFT. Thus, PFT substrate specificities reflect differences in catalytic turnover rate rather than differences in binding affinity. The catalytic constants (k_cat_/K_m_) for Arabidopsis PGGT1, which are shown in Table 2, confirm the results shown in Figures 4 and 5. Both data sets demonstrate the following substrate preferences for Arabidopsis PGGT1 (normalized to 1.0 for GFP-BD-CVIL): GFP-BD-CVIL (1.0), GFP-BD-CVII (0.14), GFP-BD-CVIM (0.07) and GFP-BD-CVIQ (0.07). Moreover, while different CaaX substrates have slightly different K_m _values, they have dramatically different k_cat _values. Indeed, the significantly higher k_cat _value for GFP-BD-CVIL is the primary determinant of substrate specificity for Arabidopsis PGGT1. While these k_cat _values can be compared, they are nevertheless low, suggesting that only a fraction of the purified PGGT1 protein was catalytically active.

**Table 1:** Kinetic constants for recombinant Arabidopsis PFT*

PFT substrate	K_m _(μM)	Specific Activity (pmol min^-1 ^mg^-1^)	k_cat _(hr^-1^)	k_cat_/K_m_ (μM^-1 ^hr^-1^)	n
GFP-BD-CVIM	5.4 +/- 0.6	5200 +/- 700	28.8 +/- 4.1	5.3 +/- 1.0	8
GFP-BD-CVIQ	5.5 +/- 0.9	8900 +/- 1900	49.1 +/- 10.5	8.9 +/- 2.4	8
GFP-BD-CVII	6.9 +/- 1.0	2300 +/- 700	12.8 +/- 3.7	1.9 +/- 0.6	8
GFP-BD-CVIL	7.0 +/- 1.5	670 +/- 140	3.7 +/- 0.8	0.5 +/- 0.2	8

Standard errors are given.

*PFT assays were performed in the presence of [1-^3^H]farnesyl diphosphate.

**Table 2:** Kinetic constants for recombinant Arabidopsis PGGT1*

PGGT1 substrate	K_m _(μM)	Specific Activity (pmol min^-1 ^mg^-1^)	k_cat _(hr^-1^)	k_cat_/K_m_ (μM^-1 ^hr^-1^)	n
GFP-BD-CVIL	17.2 +/- 4.4	110 +/- 30	0.50 +/- 0.12	0.029 +/- 0.010	7
GFP-BD-CVII	19.0 +/-14.4	17 +/- 15	0.08 +/- 0.07	0.004 +/- 0.005	4
GFP-BD-CVIQ	3.5 +/- 0.6	1.7 +/- 0.2	0.008 +/- 0.001	0.002 +/- 0.001	8
GFP-BD-CVIM	5.8 +/- 1.0	2.6 +/- 0.3	0.012 +/- 0.001	0.002 +/- 0.001	8

Standard errors are given.

*PGGT1 assays were performed in the presence of [1-^3^H]geranylgeranyl diphosphate.

Purified recombinant Arabidopsis PFT and PGGT1 were also analyzed with respect to isoprenyl diphosphate specificity. As shown in Table 3, K_m _values for FPP and GGPP are an order of magnitude lower than K_m _values for CaaX substrates in the presence of recombinant Arabidopsis PFT and PGGT1. However, as with CaaX substrates, the different specificities of Arabidopsis PFT and PGGT1 for isoprenyl diphosphates cannot be explained by differences in K_m_. Thus, the preferences of PFT and PGGT1 for different isoprenyl diphosphate substrates is primarily determined by reactivity rather than binding affinity.

**Table 3:** K_m _values (in μM) for isoprenyl diphosphates in the presence of Arabidopsis PFT and PGGT1*

Prenyltransferase	FPP	GGPP	n
PFT	0.2 +/- 0.1	0.7 +/- 0.3	7
PGGT1	0.6 +/- 0.5	0.8 +/- 0.1	7

Standard errors are given.

*PFT assays were performed in the presence of GFP-BD-CVIQ and PGGT1 assays were performed in the presence of GFP-BD-CVIL.

## Discussion

In this report, it is shown that recombinant Arabidopsis PFT exhibits broad specificity for CaaX substrates with Gln, Met, Ser, Cys, Ala, Ile, or Leu in the terminal 'X' position, whereas PGGT1 exhibits strict specificity for CaaX substrates ending in Leu. Both PFT and PGGT1 exhibit little or no specificity for the a_1 _position of the Ca_1_a_2_X motif, which is consistent with previous observations using mammalian prenyltransferases that the a_1 _position is solvent exposed and not constrained by active site amino acids [51-55]. In contrast, both prenyltransferases exhibit specificity for the a_2 _position of the Ca_1_a_2_X motif. While the mechanism for CaaX specificity remains unknown for Arabidopsis PFT and PGGT1, it is clear that the substrate specificities of both prenyltransferases reflect differences in catalytic turnover rates rather than differences in K_m _values (Tables 1 and 2). This finding suggests that, while binding affinities of GFP-BD-CVIQ, GFP-BD-CVIM, GFP-BD-CVII, and GFP-BD-CVIL to the active sites of PFT and PGGT1 are similar, the terminal amino acid of the CaaX motif dramatically affects catalysis. Moreover, the data described above provide an explanation for *era1 *and *ggb *phenotypes. PFT prenylates a wide range of CaaX substrates and compensates almost fully for loss of PGGT1 in *ggb *plants. However, PGGT1 specifically prenylates CaaL substrates and only partially compensates for loss of PFT in *era1 *plants. These biochemical differences potentially account for the mild phenotype of *ggb *mutants and the dramatic phenotype of *era1 *mutants.

The different isoprenoid specificities of Arabidopsis PFT and PGGT1 cannot be explained by differences in K_m_. Indeed, the K_m _for FPP was only slightly lower than that for GGPP in the presence of recombinant Arabidopsis PFT, despite the fact that CaaX farnesylation was 50-fold greater than geranylgeranylation in the presence of this enzyme (Figures 2 and 3). Moreover, the K_m _values for FPP and GGPP were almost identical in the presence of recombinant Arabidopsis PGGT1, despite the fact that PGGT1 catalyzed CaaX geranylgeranylation 4-fold more efficiently than farnesylation. Thus, the primary determinant of isoprenoid substrate specificity is reactivity rather than binding affinity.

The results in Figure 6 raise an interesting question. Given the higher specific activity and lower CaaX substrate specificity of PFT, why are CaaX substrates with leucine in the terminal position predominantly geranylgeranylated rather than farnesylated *in planta *[12,50]? The results in Figure 6 suggest that a CAIL (or CVIL) protein should be predominantly farnesylated *in planta *because farnesylation of CAIL (or CVIL) by *Arabidopsis *PFT is approximately 20% as efficient as farnesylation of CAIM (or CVIM), which greatly exceeds the efficiency of CAIL (or CVIL) geranylgeranylation by PGGT1. We propose that PFT is regulated *in planta*, perhaps by post-translational modifications or protein-protein interactions, to reduce recognition and farnesylation of CaaX substrates with leucine in the terminal position. Nevertheless, it is likely that CaaX substrates with leucine in the terminal position are, to some extent, farnesylated by PFT and that these aberrantly farnesylated proteins retain full or partial function. This explains why *ggb *mutants, which were expected to exhibit severe meristem and tip-growth defects due to loss of ROP function, do not exhibit these phenotypes [12,22,23].

We propose that PGGT1 activity is higher *in planta *than the purified recombinant, *E. coli*-expressed PGGT1 activity we have characterized. The activity observed with purified recombinant PGGT1 was low, suggesting that only a portion of the purified PGGT1 enzyme was active. Despite this, the relative k_cat_/K_m _values obtained for different CaaX substrates in the presence of PGGT1 were consistent with the results shown in Figures 4 and 5.

## Conclusions

In this report, recombinant Arabidopsis PFT is shown to prenylate CaaX substrates with little specificity for the terminal amino acid. In contrast, recombinant Arabidopsis PGGT1 is shown to exclusively prenylate CaaX substrates with leucine in the terminal position. These different substrate specificities provide a straightforward explanation for the phenotypes of *era1 *and *ggb *mutant plants. In addition, substrate specificities for PFT and PGGT1 are shown to reflect differences in catalytic turnover rates rather than differences in substrate binding.

## Methods

### RNA isolation

Total RNA was isolated from wild type Arabidopsis plants (ecotype Col-0) using TRIzol^® ^Reagent according to the manufacturer's instructions (Invitrogen/Life Technologies Corp., Carlsbad, CA).

### PFT and PGGT1 expression constructs

The coding sequences of the *PLP *(At3g59380), *ERA1 *(At5g40280), and *GGB *(At2g39550) genes were amplified by reverse-transcriptase-PCR using 0.5 μg of total RNA, 5 pmol of forward primer, 5 pmol of reverse primer, and the Platinum Quantitative RT-PCR Thermoscript One-Step System (Invitrogen/Life Technologies Corp., Carlsbad, CA). RT-PCR conditions included a 20-min reverse transcription step at 50°C, followed by a 5-min pre-soak at 95°C, and 25-35 cycles of the following PCR program: 95°C, 30 sec; 55°C, 30 sec; 72°C, 90 sec. A post-soak was performed at 72°C for 7 min to ensure complete product synthesis. RT-PCR products were resolved by agarose gel electrophoresis and visualized by ethidium bromide staining. The primers used for RT-PCR were as follows: PLP-CDS-forward: 5'-cac gga tcc acc atg gat tac aag gat gac gac gat aag aat ttc gac gag acc gtg cca-3'; PLP-CDS-reverse: 5'-cac gga tcc tca aat tgc tgc cac tgt aat ctt g-3'; ERA1-CDS-forward: 5'-cac gga tcc acc atg cac cac cat cac cat cac cca gta gta acc cgc ttg att-3'; ERA1-CDS-reverse: 5'-cac gga tcc tca tgc tgc ttt aaa gaa gaa ctc-3'; GGB-CDS-forward: 5'-cac gga tcc acc atg cat cat cat cat cat cat tca gag acc gcc gtg tca atc-3'; GGB-CDS-reverse: 5'-cac gga tcc tca aat tcc cgg ggc tgc aag aa-3'. RT-PCR products were confirmed by DNA sequence analysis and ligated into the BamH1 (*PLP*) or BglII (*ERA1*, *GGB*) sites of the pETDuet-1 vector (EMD Biosciences, Gibbstown, NJ).

### GFP-BD-CaaX mutagenesis

GFP-BD-CaaX expression constructs [50] were mutagenized using the QuikChange II site-directed mutagenesis kit from Stratagene (La Jolla, CA) to generate the following CaaX motifs: CVIM, CVIS, CVIC, CVIQ, CVIA, CVIL, CVII, CAIM, CFIM, CTIM, CQIM, CEIM, CKIM, CVAM, CVFM, CVTM, CVQM, CVEM, CVKM CAIL, CFIL, CTIL, CQIL CEIL, CKIL, CVAL, CVFL, CVTL, CVQL, CVEL, CVKL, SVIL. The following primers were used for mutagenesis of the GFP-BD-CVIM template: CVII-F: 5'- cgt ggc cag aag tgc gtg atc atc taa cgg gat ccc gcc -3'; CVII-R: 5'- ggc ggg atc ccg tta gat gat cac gca ctt ctg gcc acg -3'; CVIS-F: 5'- cgt ggc cag aag tgc gtg atc tcg taa cgg gat ccc gcc -3'; CVIS-R: 5'- ggc ggg atc ccg tta cga gat cac gca ctt ctg gcc acg -3'; CVIC-F: 5'- cgt ggc cag aag tgc gtg atc tgc taa cgg gat ccc gcc -3'; CVIC-R: 5'- ggc ggg atc ccg tta gca gat cac gca ctt ctg gcc acg -3'; CAIM-F: 5'- cgt ggc cag aag tgc gcg atc atg taa cgg gat ccc gcc -3'; CAIM-R: 5'- ggc ggg atc ccg tta cat gat cgc gca ctt ctg gcc acg -3'; CFIM-F: 5'- cgt ggc cag aag tgc ttt atc atg taa cgg gat ccc gcc -3'; CFIM-R: 5'- ggc ggg atc ccg tta cat gat aaa gca ctt ctg gcc acg -3'; CTIM-F: 5'- cgt ggc cag aag tgc acg atc atg taa cgg gat ccc gcc -3'; CTIM-R: 5'- ggc ggg atc ccg tta cat gat cgt gca ctt ctg gcc acg -3'; CQIM-F: 5'- cgt ggc cag aag tgc cag atc atg taa cgg gat ccc gcc -3'; CQIM-R: 5'- ggc ggg atc ccg tta cat gat ctg gca ctt ctg gcc acg -3'; CEIM-F: 5'- cgt ggc cag aag tgc gag atc atg taa cgg gat ccc gcc -3'; CEIM-R: 5'- ggc ggg atc ccg tta cat gat ctc gca ctt ctg gcc acg -3'; CKIM-F: 5'- cgt ggc cag aag tgc aag atc atg taa cgg gat ccc gcc -3'; CKIM-R: 5'- ggc ggg atc ccg tta cat gat ctt gca ctt ctg gcc acg -3'; CVAM-F: 5'- cgt ggc cag aag tgc gtg gcc atg taa cgg gat ccc gcc -3'; CVAM-R: 5'- ggc ggg atc ccg tta cat ggc cac gca ctt ctg gcc acg -3'; CVFM-F: 5'- cgt ggc cag aag tgc gtg ttc atg taa cgg gat ccc gcc -3'; CVFM-R: 5'- ggc ggg atc ccg tta cat gaa cac gca ctt ctg gcc acg -3'; CVTM-F: 5'- cgt ggc cag aag tgc gtg acc atg taa cgg gat ccc gcc -3'; CVTM-R: 5'- ggc ggg atc ccg tta cat ggt cac gca ctt ctg gcc acg -3'; CVQM-F: 5'- cgt ggc cag aag tgc gtg cag atg taa cgg gat ccc gcc -3'; CVQM-R: 5'- ggc ggg atc ccg tta cat ctg cac gca ctt ctg gcc acg -3'; CVEM-F: 5'- cgt ggc cag aag tgc gtg gag atg taa cgg gat ccc gcc -3'; CVEM-R: 5'- ggc ggg atc ccg tta cat ctc cac gca ctt ctg gcc acg -3'; CVKM-F: 5'- cgt ggc cag aag tgc gtg aag atg taa cgg gat ccc gcc -3'; CVKM-R: 5'- ggc ggg atc ccg tta cat ctt cac gca ctt ctg gcc acg -3'. The following primers were used for mutagenesis of the GFP-BD-CVIL template: CVIQ-F: 5'- cgt ggc cag aag tgc gtg atc cag taa cgg gat ccc gcc -3'; CVIQ-R: 5'- ggc ggg atc ccg tta ctg gat cac gca ctt ctg gcc acg -3'; CVIA-F: 5'- cgt ggc cag aag tgc gtg atc gcg taa cgg gat ccc gcc -3'; CVIA-R: 5'- ggc ggg atc ccg tta cgc gat cac gca ctt ctg gcc acg -3'; CAIL-F: 5'- cgt ggc cag aag tgc gcg atc ctg taa cgg gat ccc gcc -3'; CAIL-R: 5'- ggc ggg atc ccg tta cag gat cgc gca ctt ctg gcc acg -3'; CFIL-F: 5'- cgt ggc cag aag tgc ttt atc ctg taa cgg gat ccc gcc -3'; CFIL-R: 5'- ggc ggg atc ccg tta cag gat aaa gca ctt ctg gcc acg -3'; CTIL-F: 5'- cgt ggc cag aag tgc acg atc ctg taa cgg gat ccc gcc -3'; CTIL-R: 5'- ggc ggg atc ccg tta cag gat cgt gca ctt ctg gcc acg -3'; CQIL-F: 5'- cgt ggc cag aag tgc cag atc ctg taa cgg gat ccc gcc -3'; CQIL-R: 5'- ggc ggg atc ccg tta cag gat ctg gca ctt ctg gcc acg -3'; CEIL-F: 5'- cgt ggc cag aag tgc gag atc ctg taa cgg gat ccc gcc -3'; CEIL-R: 5'- ggc ggg atc ccg tta cag gat ctc gca ctt ctg gcc acg -3'; CKIL-F: 5'- cgt ggc cag aag tgc aag atc ctg taa cgg gat ccc gcc -3'; CKIL-R: 5'- ggc ggg atc ccg tta cag gat ctt gca ctt ctg gcc acg -3'; CVAL-F: 5'- cgt ggc cag aag tgc gtg gcc ctg taa cgg gat ccc gcc -3'; CVAL-R: 5'- ggc ggg atc ccg tta cag ggc cac gca ctt ctg gcc acg -3'; CVFL-F: 5'- cgt ggc cag aag tgc gtg ttc ctg taa cgg gat ccc gcc -3'; CVFL-R: 5'- ggc ggg atc ccg tta cag gaa cac gca ctt ctg gcc acg -3'; CVTL-F: 5'- cgt ggc cag aag tgc gtg acc ctg taa cgg gat ccc gcc -3'; CVTL-R: 5'- ggc ggg atc ccg tta cag ggt cac gca ctt ctg gcc acg -3'; CVQL-F: 5'- cgt ggc cag aag tgc gtg cag ctg taa cgg gat ccc gcc -3'; CVQL-R: 5'- ggc ggg atc ccg tta cag ctg cac gca ctt ctg gcc acg -3'; CVEL-F: 5'- cgt ggc cag aag tgc gtg gag ctg taa cgg gat ccc gcc -3'; CVEL-R: 5'- ggc ggg atc ccg tta cag ctc cac gca ctt ctg gcc acg -3'; CVKL-F: 5'- cgt ggc cag aag tgc gtg aag ctg taa cgg gat ccc gcc -3'; CVKL-R: 5'- ggc ggg atc ccg tta cag ctt cac gca ctt ctg gcc acg -3'. All GFP-BD-CaaX constructs were confirmed by DNA sequence analysis.

### Protein expression and purification

Cultures of *E. coli *Rosetta cells (EMD Biosciences, Gibbstown, NJ) containing PFT, PGGT1, or GFP-BD-CaaX expression constructs were grown to log phase (A_600 _= 0.7-0.8) in Luria Broth containing 100 μg ml^-1 ^ampicillin. Recombinant protein expression was then induced with 1 mM IPTG (PFT and PGGT1) or 0.1% L-arabinose (GFP-BD-CaaX proteins) at 20°C for 16 hr. After centrifugation at 16,000*g *for 2 min, cell pellets were resuspended in 1 ml of STE buffer (150 mM NaCl, 10 mM Tris-HCl, pH 7.5, 1 mM EDTA) containing Complete Protease Inhibitors (Roche Diagnostics, Indianapolis, IN) and cells were disrupted by vigorous agitation in the presence of glass beads. Cell extracts were cleared by centrifugation at 16,000*g *for 2 min at 4°C. Protein purification was accomplished at 4°C in three steps: 1) PD-10 gel filtration chromatography in 50 mM sodium phosphate, pH 7.0, 0.3 M NaCl [to remove EDTA], 2) immobilized metal affinity chromatography [IMAC] using Talon^® ^Co^2+^-based resin (Clontech, Mountain View, CA) according to the manufacturer's instructions, and 3) PD-10 gel filtration chromatography in 50 mM sodium phosphate, pH 7.0, 0.3 M NaCl [to remove imidazole, which inhibits protein prenylation].

### *In vitro *prenylation assays

*In vitro *prenylation reactions contained protein prenyltransferase (100 μg of *E. coli *extract containing recombinant PFT or PGGT1, 0.005 μg of purified PFT, or 0.5 μg of purified PGGT1), 5-40 μg of GFP-BD-CaaX protein, and either [1-^3^H]farnesyl diphosphate (26.2 Ci/mmol, Perkin Elmer, Waltham, MA) or [1-^3^H]geranylgeranyl diphosphate (19.5 Ci/mmol, Perkin Elmer, Waltham, MA) in 125 μl of 50 mM Hepes (pH 7.5), 20 mM MgCl_2_, 5 mM DTT, 0.1% Zwittergent, and 5 mM ZnCl_2_. Reactions were incubated at 30°C for 30 min, after which two 50-μl portions were terminated in 950 μl of 1 M ethanolic HCl. Precipitated proteins were collected on GF/A glass fiber filters (Whatman, Piscataway, NJ), washed with 10 ml of 95% ethanol, and quantified by liquid scintillation using BioSafe II cocktail (RPI Corporation, Mt. Prospect, IL). A 25 μl portion of each reaction was resolved by SDS-PAGE and prenylated proteins were visualized using Amplify fluorographic reagent (GE Healthcare, Piscataway, NJ) and Kodak AR5 film (Eastman Kodak, Rochester, NY).

## Authors' contributions

MA performed site-directed mutagenesis on GFP-BD-CaaX proteins, protein expression and purification (i.e., PFT, PGGT1 and GFP-BD-CaaX proteins), and the kinetic assays described in Figures 7 and 8 and Tables 1, 2 and 3. DHH created the *PLP *and *PLP *+ *GGB *expression constructs. DNC designed all experiments, created the *PLP *+ *ERA1 *expression construct and performed the experiments shown in Figures 2, 3, 4, 5 and 6. All authors read and approved the final manuscript.
